# Resources and Readmission for COPD Exacerbation in Pneumology Units in Spain: The COPD Observatory Project

**DOI:** 10.3390/healthcare13030317

**Published:** 2025-02-04

**Authors:** Myriam Calle Rubio, Pilar Cebollero Rivas, Cristóbal Esteban, Antonia Fuster Gomila, José Alfonso García Guerra, Rafael Golpe, Jesús R. Hernández Hernández, Jessica Sara Lozada Bonilla, Juan Marco Figueira-Gonçalves, Eduardo Marquez, José Javier Martínez Garceran, Javier de Miguel-Díez, Ana Pando-Sandoval, Juan A. Riesco, Salud Santos Pérez, Rafael Sánchez-del Hoyo, Juan Luis Rodríguez Hermosa

**Affiliations:** 1 Pulmonology Department, Hospital Clínico San Carlos, Department of Medicine, School of Medicine, Instituto de Investigación Sanitaria del Hospital Clínico San Carlos (IdISSC), Universidad Complutense de Madrid, 28040 Madrid, Spain; mcal01@ucm.es; 2 CIBER de Enfermedades Respiratorias (CIBERES), 28040 Madrid, Spainsaludsantos@bellvitgehospital.cat (S.S.P.); 3 Respiratory Department, University Hospital of Navarra (HUN), 31008 Pamplona, Spain; pilar62rivas@gmail.com; 4 Respiratory Department, BioCruces-Bizkaia Health Research Institute, Hospital Universitario Galdakao-Usansolo, Health Services Research on Chronic Patients Network (REDISSEC), Research Network on Chronicity, Primary Care and Health Promotion (RICAPPS), 48960 Galdakao, Spain; 5 Respiratory Medicine Department, Hospital Universitario Son Llàtzer, 07198 Palma de Mallorca, Spain; 6 Respiratory Medicine Service, Hospital La Mancha Centro de Alcázar de San Juan, 13600 Ciudad Real, Spain; fonsus_14@hotmail.com; 7 Pneumology Department, Hospital Universitario Lucus Augusti, 27003 Lugo, Spain; 8 Respiratory Department, Ávila Health Care Complex, 05071 Ávila, Spain; jhernandez@separ.es; 9 Consorci Hospital General Universitari, 46014 Valencia, Spain; 10 University Hospital Nuestra Señora de Candelaria, 38010 Santa Cruz de Tenerife, Spain; 11 UGC of Respiratory Diseases, University Hospital Virgen del Rocío, Institute of Biomedicine of Seville (IBIS), University of Seville, 41013 Sevilla, Spain; 12 Respiratory Diseases Department, Santa Lucia University Hospital, 30202 Cartagena, Spain; josejmg@hotmail.com; 13 Respiratory Department, Gregorio Marañón General University Hospital, Faculty of Medicine, Gregorio Marañón Biomedical Research Institute, Complutense University of Madrid, 28007 Madrid, Spain; 14 Respiratory Medicine Department, Hospital Universitario Central de Asturias, 33011 Oviedo, Spain; 15 Respiratory Department, San Pedro de Alcántara University Hospital, 29670 Cáceres, Spain; 16 Pulmonology Department, Pneumology Research Group, Institut d’Investigació Biomèdica de Bellvitge—IDIBELL, Universitat de Barcelona, L’Hospitalet de Llobregat, 08907 Barcelona, Spain; 17 Research Methodological Support Unit and Preventive Department, Hospital Clínico San Carlos, IdISSC, 28040 Madrid, Spain; rafaelsanchezdelhoyo@gmail.com

**Keywords:** hospital admission, COPD, resources, healthcare policy, health disparities, healthcare quality

## Abstract

Chronic obstructive pulmonary disease (COPD) represents one of the most frequent causes of hospital readmissions and in-hospital mortality. One in five patients requires readmission within 30 days of discharge following an admission for exacerbation. These ‘early readmissions’ increase morbidity and mortality, as patients often do not recover their baseline lung function. The identification of factors associated with increased risk has been a major focus of research in recent years. Studies describe patient-related predictors, although some studies also suggest that better-resourced centres provide superior care. **Objective**: To describe resources, performance, and care provided in pneumology units in Spain, assessing their association with 30-day readmission for COPD and in-hospital mortality. **Methods**: This survey was conducted in 116 hospitals responsible for the COPD pathway in pneumology units/departments from November 2022 to March 2023. **Results**: Of the 116 participating hospitals, 56% had a pneumology department while 25.9% had a pneumology section. The vast majority were public and university hospitals. The number of beds allocated to pneumology/100,000 inhabitants was 6.6 (3.1–9.2) and pulmonologist staffing was 3.3 (2.6–4.1) per 100,000 inhabitants. There was an intermediate respiratory care unit (IMCU) dependent on the pneumology department in 31.9% of units and a respiratory team for 24 h emergency care in 30% of units, while only 9.5% had interventional pneumology units for bronchoscopic procedures. COPD rehabilitation programmes were offered in 58.6% of pneumology units. The average rate of patients on ventilatory support in acute failure was 13.8 (9.2–25) per 100 discharges, with a 30-day COPD readmission rate of 14.9%, with significant differences according to the level of complexity (*p* = 0.041), with a mean length of stay of 8.72 (1.26) days. The overall in-hospital mortality in pneumology units was 4.10 (1.18) per 100 admissions. In the adjusted model, having a discharge support programme and interventions performed during admission (number of patients with ventilatory support) were predictors of a favourable outcome. Hospital stay was also maintained as a predictor of an unfavourable outcome. **Conclusions**: There is significant variability in resources and the organisation of care in pneumology units in Spain. The availability of a discharge support programme and greater use of ventilatory support at discharge are factors associated with a lower 30-day COPD readmission rate in the pneumology unit. This information is relevant to improve the care of patients with COPD and as a future line of research.

## 1. Introduction

Chronic obstructive pulmonary disease (COPD) represents one of the most prevalent causes of hospital admissions and medical consultations [1,2], posing significant social and economic burdens that are anticipated to rise in the coming years [3,4,5]. Patients with COPD frequently encounter exacerbation episodes necessitating hospitalisation. Among those surviving hospitalisation, readmission due to acute exacerbation shortly after discharge remains a critical, unresolved issue. The high readmission rates associated with COPD correlate with increased mortality risk and substantial financial strain. Therefore, the prevention and reduction of readmissions have been prioritised as crucial management strategies. Recent research has delved into readmission rates and post-discharge risk factors. Audit studies have highlighted varied outcomes in COPD management, which are contingent upon patient characteristics and the medical care received [6,7], as evidenced in numerous studies examining the influence of treatment regimens and hospital resources on hospital mortality and readmissions post-discharge for COPD exacerbation [8,9,10].

The European COPD audit revealed significant variability in resourcing and care organisation across European hospitals treating COPD exacerbations. While some studies suggest that better-resourced facilities provide superior care [11,12], greater resources in larger hospitals does not invariably guarantee improved access to or standards of care [13]. Hence, other aspects of the care process, such as units led by qualified staff and standardised management plans to reduce practise variability, are essential elements upon which the quality of clinical care is founded.

Clinical audits are essential tools for identifying deficiencies in care, thereby raising awareness of these issues and ultimately improving the quality of care. Since 2014, the Spanish Society of Pneumology and Thoracic Surgery (SEPAR) has spearheaded a process of auditing COPD care, with the objective of using clinical audits as a mechanism for continuous quality improvement [7,14]. In 2015, the RECALAR project [15], funded by SEPAR, assessed the resources and organisational structure of respiratory disease care in the pneumology units of Spanish National Health System hospitals. Currently, there is a paucity of information regarding resources and organisational models for COPD care within pneumology units. Against this backdrop, the COPD Observatory project was conceived with the aim of analysing the resources, activities, and organisational models in COPD care within Spanish pneumology units/departments through a survey conducted by clinical managers. Additionally, the project seeks to examine the association of these factors with outcomes such as 30-day COPD readmission rates and overall in-hospital mortality in pneumology units.

## 2. Materials and Methods

The COPD Observatory project was conceived as a cross-sectional survey assessing the resources and organisation dedicated to COPD care, promoted by SEPAR. The Spanish Society of Pneumology and Thoracic Surgery extended an official invitation to participate in the study to all respiratory units within Spanish National Health System hospitals, as per the Ministry of Health’s 2021 registry [16]. Out of 223 hospitals invited, 116 (52%) participated. The intended respondents for the survey were those responsible for the COPD pathway in pneumology units/departments. The fieldwork was conducted from November 2022 to March 2023. The participating hospitals and investigators are detailed in Supplementary S1.

The project’s steering committee comprised 17 chest physicians experienced in COPD, representing each of the 17 regional respiratory societies in Spain. Database items were selected by the steering committee and discussed via email and in face-to-face meetings. The survey included distinct questions regarding hospital and respiratory unit resources. A detailed list of the survey items is provided in Supplementary S2. Data sources included a survey of pneumology units regarding resources and care (level of hospital complexity, number of pulmonologists and nurses, number of pneumology beds, complexity of the bronchoscopy unit, availability of an intermediate respiratory care unit, 24 h emergency care, a follow-up and discharge support programme for COPD, and rehabilitation programme) and performance (number of spirometries, length of stay, number of discharges, number of patients in a home ventilation programme and receiving non-invasive acute ventilatory support), as well as the number of 30-day COPD readmissions and overall mortality in pneumology units referred from data analysis for the year 2022 through coding of discharge reports by hospital admission departments. We analysed the data and results of the care provided to patients who were treated in these pneumology departments during the year 2022.

Data were remotely entered by each participating site onto a centrally controlled server. The web tool offered a help service with explanatory text to facilitate survey question interpretation. Surveys were administered after identifying the centres and contact persons at the 116 participating sites. For additional quality control during data collection, all completed surveys were reviewed and regular reviews of the database records were carried out to identify any issues or inconsistencies.

### Statistical Analysis

Qualitative variables are presented with their frequency distribution, while quantitative variables are summarised by their mean and standard deviation (SD). Quantitative variables exhibiting an asymmetric distribution are summarised using the median and interquartile range (IQR). For the comparison of qualitative variables, the χ2 test or Fisher’s exact test was employed when necessary. Comparisons of means between two independent groups were conducted using Student’s t-test if the variables followed a normal distribution, or the nonparametric Mann–Whitney U test for asymmetric variables. To compare means across more than two independent groups, analysis of variance (ANOVA) was used, or the nonparametric Kruskal–Wallis test for asymmetric variables. To examine the correlation between quantitative variables, the Pearson correlation coefficient was utilised, or Spearman’s correlation coefficient when appropriate.

A simple linear regression model was applied to explore the association between the number of total admissions per 100 discharges in 2022 and potential individually associated factors. Additionally, a multiple linear regression model was employed to study the association jointly. A significance level of 5% was accepted for all tests. Data processing and analysis were performed using IBM SPSS Statistics v.26 and R v.4.4.1 software.

## 3. Results

### 3.1. Hospital Resources

**Figure 1:**
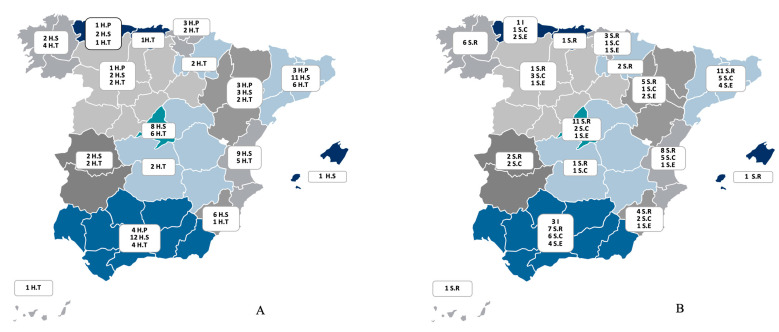
(**A**) The distribution of the participating centres across the 16 regions of Spain (indicated by colour) according to the level of complexity of the hospital centre: level I, or primary hospital (H.P); level II, or secondary hospital (H.S); level III, or tertiary hospital (H.T). Data are presented as numbers. (**B**) The distribution of the participating centres across the 16 regions of Spain (indicated by colour) according to pneumology organisational entity: I (institute or area of clinical management); S.R (department); S.C (section); S.E: no entity. Data are presented as numbers.

The characteristics of the participating hospitals, categorised by their level of complexity, are summarised in Table 1. The majority of the participating centres were public institutions, with approximately three-quarters identified as university or teaching hospitals. Notably, 56% of these hospitals had a dedicated pneumology department, while 14.7% lacked an organisational structure for pneumology. The distribution of participating centres across the various autonomous communities, based on the hospital’s level of complexity and the type of organisational entity of the pneumology unit, is depicted in Figure 1.

### 3.2. Respiratory Unit Resources

The resources of the respiratory units are summarised in Table 1. On average, pneumology units had 6.6 beds (range 3.1–9.2) per 100,000 inhabitants. The median number of pulmonologists per 100,000 inhabitants was 3.3 (range 2.6–4.1), with variations between low-complexity centres at 2.9 (range 2.3–3.9) and high-complexity centres at 3.7 (range 3–4.6). A majority of high-complexity centres had an intermediate care unit (IMCU) (65%) and offered 24 h urgent care staffed by pulmonologists (69.8%). More than half of the centres (58.6%) had rehabilitation programmes, with 54.4% offering both inpatient and home rehabilitation. A minority of centres (9.5%) provided endoscopic volume reduction techniques for patients with COPD. Specialised COPD consultations were available in 52.6% of the units, follow-up and discharge support programmes in 55.2%, immediate COPD care devices in 44%, a specialist consultant for the COPD process in 45.6%, nursing consultations in 38.8%, and smoking prevention consultations in 48.3%. These resources were more frequently available in tertiary or level III centres. Nonetheless, follow-up and support programmes at discharge were also significantly present in lower-complexity centres. 

### 3.3. Respiratory Unit Performance

The average number of hospital discharges from pneumology units was 266.2 (range 186.8–399.2) per 100,000 inhabitants, with a mean length of stay of 8.72 (SD 1.26) days. The average rate of patients requiring ventilatory support for acute failure was 13.8 (range 9.2–25) per 100 discharges, with a 30-day COPD readmission rate of 14.9%, showing significant differences according to the hospital’s level of complexity (*p* = 0.041). Overall, in-hospital mortality was 4.10 (SD 1.18) per 100 admissions to pneumology units, with no significant differences based on the hospital’s level of complexity. The average number of patients in home ventilation programmes was 57.8 (range 20.3–100.5) per 100,000 inhabitants. The average rate of spirometries performed per month per 100,000 inhabitants was 121.4 (range 70–225.7), with notable variations between low-complexity centres (170.5, range 95.8–320) and high-complexity centres (141.7, range 77.7–230.7). The number of bronchoscopic procedures performed per year per 100,000 inhabitants was 164 (range 114.8–244.8). The organisational performance of the respiratory units is summarised in Table 2.

### 3.4. Factors Associated with Outcomes: Overall In-Hospital Mortality and 30-Day COPD Readmissions for COPD Exacerbations

**Figure 2:**
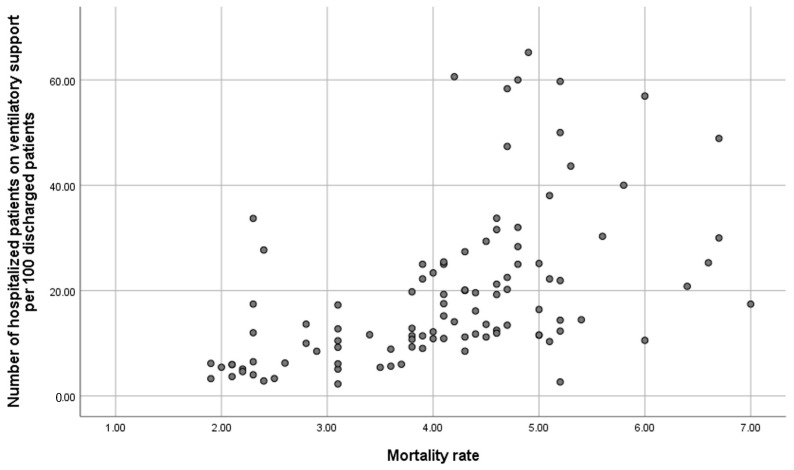
Correlation between in-hospital mortality and number of acutely ventilated patients per 100 discharges.

Table 3 outlines the bivariate association between 30-day readmissions for COPD and variables related to hospital resources, organisation, and interventions performed during admission and at discharge. Significant variables associated with a lower 30-day COPD readmission rate include having a follow-up and support programme at discharge, immediate care, specialised COPD consultation, nursing consultation, specialist consultants in the COPD process, hospital stay, and the number of patients receiving ventilatory support. Conversely, most resource and organisation variables were not related to in-hospital mortality (Supplementary S3), with a regression coefficient correlation of 0.622 between in-hospital mortality and the number of acutely ventilated patients per 100 discharges (Figure 2).

In the multivariable analysis of 30-day COPD readmissions (Table 4), the adjusted model retained only outcome predictors linked to organisation (having a follow-up and discharge support programme) and interventions performed during admission (number of patients on ventilatory support), both predictors of favourable outcomes. Hospital stay was also identified as a predictor of unfavourable outcomes.

## 4. Discussion

This study provides a significant dataset from pneumology units in Spain, detailing COPD resources, activities, and care models, as well as data on readmissions and mortality for patients hospitalised due to COPD exacerbations. It also offers insights into potential interventions to enhance the quality of care during hospital admissions. Findings indicate that resources such as discharge support programmes and interventions like increased ventilatory support at discharge are associated with a reduced risk of 30-day COPD readmissions, whereas a longer hospital stay predicts unfavourable outcomes, likely reflecting COPD or patient severity.

Despite existing COPD management guidelines, there are no recommendations for minimum resources or service organisation to ensure optimal care. This study examines the variation in the provision of resources to manage patients with COPD across 116 pneumology units in Spain, exploring the correlation between hospital size, resource provision, and outcomes such as 30-day readmission rates for COPD exacerbation and in-hospital mortality. It also updates data on the organisational structure and functioning of Spanish pneumology units since the RECALAR [15] document was published in 2017.

Our data reveal a distribution of pneumology units in Spanish hospitals similar to that of 2017. Of the population assessed, over half (56%) have an institutional department designation, 25.9% are sections, and the remainder are part of internal medicine departments. According to our analysis, Spain has 6.6 pneumology beds per 100,000 inhabitants and an average of 3.3 pulmonologists per 100,000 inhabitants. These figures align closely with the 2017 survey data [15], considering the impact of the COVID-19 pandemic declared in 2020, which underscored deficiencies in respiratory care due to high morbidity and mortality. Evidence suggests that more specialists per bed correlates with reduced hospital mortality, shorter stays, and lower readmission rates, thus improving health system efficiency. The AUDIPOC audit [12] also associates a higher number of respiratory specialists per bed with lower COPD mortality. Additionally, our analysis shows minimal variation by hospital complexity level (2.9 pulmonologists/100,000 in level I or primary hospitals versus 3.7/100,000 in level III or tertiary centres). Optimal specialist allocation per bed and comprehensive activity consideration are crucial for effective, high-quality hospital care. These findings underscore the need for improvement, especially since most surveyed centres are public and university affiliated.

Hospitalisation due to COPD exacerbations is associated with high in-hospital mortality rates, which range between 2.5 and 15%, varying based on patient characteristics and the research setting [16,17]. In our study, the overall in-hospital mortality in pneumology units was 4.1% (SD 1.1%), lower in level I centres at 2.6% (SD 1.2%), and higher in high-complexity centres, averaging 4.2% (SD 1.1%). There were no significant differences in in-hospital mortality based on the complexity level of the centres or the resources of the pneumology units. Systematic reviews and meta-analyses indicate that several characteristics of patients with COPD and comorbidities significantly correlate with increased mortality [18]. A meta-analysis of over 60,000 patients with COPD revealed that non-surviving patients had more hospitalisations in the previous year, longer hospital stays, greater dyspnoea during hospitalisation, and were more likely to require ventilatory support [19]. Data from the European AUDIPOC audit demonstrated that greater resources in larger hospitals did not necessarily guarantee better access to care. Despite having more resources, larger hospitals did not show a marked difference in guideline adherence, emphasising that optimising risk stratification upon admission for COPD exacerbation, diagnosing respiratory acidosis within the first hours of admission, and timely specialist respiratory care team involvement result in better patient outcomes [20].

In the European COPD audit, ventilatory support was significantly associated with in-hospital mortality [12]. The reasons for delaying or not offering non-invasive ventilatory support may be influenced by the availability and access to intermediate respiratory care units, as initially noted by Roberts et al. [21] and corroborated by the European COPD audit data [13]. Hence, early access to specialists who can provide non-invasive support in controlled settings, such as intermediate care units (IMCUs), and having a specialised team that allows for continuous monitoring and timely intervention in case of respiratory complications is crucial for improving outcomes in critically ill patients. In the 2012 European audit, over one-third of the centres reported they could not treat all eligible patients throughout the year for both invasive and non-invasive ventilation [13,20]. In our study, intermediate respiratory care units attached to pneumology units were present in 65% of high-complexity (level III) hospitals, with a median of 7 beds (range 6–8.7) and a nurse-to-bed ratio of 4 (range 4–6). Most of these IMCUs were established during the SARS-CoV-2 pandemic. Two-thirds of level III hospitals had 24 h emergency care provided by pulmonologists. These units, being less costly than intensive care units, improve hospital efficiency by optimally utilising resources for each level of care, thus allowing better patient flow. Studies have shown that intermediate respiratory care units reduce mortality in severe exacerbations of chronic respiratory disease by administering non-invasive ventilatory support to patients with respiratory failure, a treatment proven effective in avoiding intubation in many cases and reducing the risks associated with invasive ventilation [22]. The expansion of these units within pneumology units in Spain reflects the increasing need for intermediate respiratory care and their essential role in the current hospital structure.

Ventilatory support for acidotic respiratory failure during COPD admission has been shown to be a protective factor against in-hospital mortality [23]. Our study indicates that a median of 13.8 (range 9.2–25) inpatients per 100 discharges in pneumology units benefit from acute non-invasive ventilation, with a median of 88.2 patients on long-term non-invasive ventilation per 100,000 inhabitants. These figures have doubled compared to those reported in the RECALAR [15] report in 2017, which indicated 46 patients per 100,000 inhabitants being on home ventilatory support. These data are also somewhat higher than the European average of 66 patients per 100,000 inhabitants [24]. Home ventilation for COPD has been shown to reduce the risk of COPD readmission, especially in patients with a history of respiratory acidosis and hypercapnia [25,26]. Despite the increased use of mechanical ventilation as home therapy, information on its prescription in COPD remains limited. Data from an international web survey of physicians prescribing long-term non-invasive supportive care therapy revealed that the prescription rate of home mechanical ventilation in patients with COPD was 38.5% of all prescriptions [25]. However, there is significant variability in the frequency of home mechanical ventilation prescriptions for COPD across different countries and regions [27,28].

COPD has become one of the diseases with the highest rates of early readmission within 30 days. One in five patients requires rehospitalisation within 30 days of discharge following an admission for exacerbation [29]. A recent systematic review reported that the rate of readmissions for acute exacerbations of COPD ranged from 6% to 24% within 30 days of discharge, based on an analysis of 24 studies [30]. These measurements may be confounded by variations in metric generation. Additionally, study design, age stage, WHO region, and length of hospital stay have been identified as potential sources of readmission rate heterogeneity. In our study, the median 30-day COPD readmission rate was 14.9% (range 10.1–18.7%), being lower in level I complexity centres (10.7%, range 8.2–15%) compared to level III high-complexity centres with a median of 13.1% (range 8.6–16.7%). These “early readmissions” increase morbidity and mortality, as patients often do not recover their baseline lung function [31,32]. Additionally, readmissions after COPD hospitalisation add to the economic burden, estimated to cost $13 billion and associated with poor outcomes [23]. The identification of factors associated with increased risk has been a major focus of research in recent years. Despite the heterogeneity of studies, the most frequently described predictive variables related to the patient include advanced age, comorbidities, low socioeconomic status, social situation, previous admissions, severity of illness, low adherence to treatment, the need for home ventilation, and fragility [33,34]. Additionally, factors related to the health system, such as hospital stay, lack of a defined follow-up programme, and poor health education, are also significant. Our observation indicated that longer hospital stays were associated with higher readmission rates, consistent with systematic reviews demonstrating that length of stay correlates with an increased risk of readmissions for COPD [33]. In addition, our analysis found that the 30-day readmission rate was closely related to the availability of a follow-up and support programme after the acute phase of the disease. Studies evaluating comprehensive programmes that provide multidisciplinary care from hospital to home have shown mixed results [35,36]. Approximately 50% of these readmissions could be avoided, often resulting from a fragmented healthcare system that discharges patients prematurely, poorly plans follow-up care transitions, inadequately communicates discharge instructions, fails in family education about the disease, and lacks communication with outpatient physicians responsible for future care [37]. However, it is important to recognise that there is a subset of patients with COPD who are frail, with associated social factors, lacking family support, and with multiple comorbidities. These ‘hospital-dependent patients’ represent a vulnerable population with chronic clinical conditions. Recent studies suggest that traditional resources or interventions have not effectively reduced readmissions in this high-risk group [38,39,40]. There is a pressing need to continue improving community health services for more vulnerable patients, focusing on care planning and research to identify the most vulnerable patients with COPD early.

### 4.1. Future Perspectives

The results of our study indicate that the availability of follow-up and discharge support programmes is associated with a lower readmission rate in patients with COPD. This is a healthcare resource that should be prioritised by identifying patients at higher risk of readmission. Research into artificial intelligence-based predictive tools can help predict 30-day readmission risk in hospitalised patients prior to discharge, allowing early implementation of personalised clinical care plans more efficiently. A recent study showed that an artificial neural network (ANN) model achieved a significant 48% reduction in readmission rates [41]. Furthermore, standardisation of hospital care with early and correct indication of ventilatory support use in a more controlled setting alongside further research into the use of non-invasive ventilation in the post-hospital management of patients with COPD may be efficient strategies to achieve an improvement in the quality of care focused on reducing readmission and mortality rates in COPD.

### 4.2. Study Limitations

Although this study provides new information and has a broad scope, to accurately interpret our results, several considerations must be taken into account. The information sources are reported by unit managers and have not been independently verified. Nonetheless, previous research studies on this topic, such as the 2017 RECALAR survey [15], were conducted using the same methodology, enabling comparison with results from over five years ago. It is worth noting that data collection was performed via an electronic form with explanatory texts to minimise ambiguity and interpretation issues. Data on outcomes (mortality and 30-day readmissions for COPD) and activities performed by the pneumology unit were gathered from the admission departments at the centres after coding hospitalisation reports. However, these measurements may vary due to differences in metric generation across centres, such as variations in the use of the International Classification of Diseases, and different exclusion criteria, such as transfers to other hospitals, which limit comparisons between centres.

## 5. Conclusions

This study has revealed significant variations in resources and performance according to the level of complexity. It highlights a stagnation in the growth of human resources, with an average of 3.3 pulmonologists per 100,000 inhabitants, and an increase in intermediate respiratory care units (IMCUs), present in 65% of pneumology departments in high-complexity centres, with an increase in the use of mechanical ventilation as home therapy. The median 30-day COPD readmission rate was 14.9%, identifying resources such as discharge support programmes and interventions like increased ventilatory support at discharge as factors associated with a reduced risk of readmission for COPD, while a longer hospital stay was an unfavourable predictor. There were no significant differences in in-hospital mortality based on the complexity level of the centres or the resources of the pneumology units. This information provides the opportunity to plan actions that will improve the quality of care and identify areas for improvement and future lines of research.

## Figures and Tables

**Table 1:** Characteristics of participating hospitals, resources available, and care provided in units.

	Overall	Level I	Level II	Level III
Number of centres, n (%)	116	15 (12.9)	58 (50)	43 (37.1)
Number of inhabitants in reference population, median (IQR)	170,000 (49,560–30,3750)	95,000 (23,750–138,750)	160,000 (31,552–282,500)	300,000 (48,000–437,406)
Number of hospital beds per 100,000 inhabitants, median (IQR)	157 (125–230.3)	146.4 (100–189.7)	147 (114.8–221.8)	175.3 (134.8–248)
University hospital, n (%)	89 (76.7)	3 (20)	44 (75.9)	42 (97.7)
Public hospital, n (%)	113 (97.4)	14 (93.3)	58 (100)	41 (95.3)
Institutional denomination of the pneumology department or unit, n (%)				
– Institute – Department – Section – No organisational entity	4 (3.4) 65 (56) 30 (25.9) 17 (14.7)	3 (20) 3 (20) 9 (60)	2 (3.4) 25 (43.1) 23 (39.7) 8 (13.8)	2 (4.7) 37 (86) 4 (9.3) 0
Number of pulmonologists/100,000 inhabitants, median (IQR)	3.3 (2.6–4.1)	2.9 (2.3–3.9)	3 (2.4–4)	3.7 (3–4.6)
Number of resident interns in pneumology, median (IQR)	0 (2–4)	0 (0–0)	0 (0–4)	6 (4–8)
Number of pneumology beds per 100,000 inhabitants, median (IQR)	6.6 (3.1–9.2)	0 (0–5.9)	6.2 (0–9.5)	7.5 (5.5–9.6)
Number of pneumology beds with telemetry, median (IQR)	0 (0–6)	0	0 (0–4)	6 (0–8)
Intermediate care unit (IMCU) managed by pulmonologists, n (%)	37 (31.9)	0	9 (15.5)	28 (65.1)
Number of beds in IMCU, median (IQR)	6 (4–8)	0	5.1 (4–6.5)	7 (6–8.7)
Ratio of nurses to beds in IMCU *, median (IQR)	4 (4–6)	-	5.1 (4–5)	5 (4–6)
24 h emergency care provided by pulmonologists, n (%)	35 (30.2)	0	5 (8.6)	30 (69.8)
– Onsite	26 (74.3)		5 (100)	21 (70)
Pulmonary rehabilitation programme for COPD n (%)	68 (58.6)	4 (26.7)	33 (56.9)	31 (73.8)
Type of programme				
– Hospital – Community-based – Mixed	27 (39.7) 4 (5.9) 37 (54.4)	4 (100)	15 (45.5) 1 (3) 17 (51.5)	12 (38.7) 3 (9.7) 16 (51.6)
Exercise test with oxygen uptake, n (%)	47 (40.5)	2 (13.3)	12 (20.7)	33 (76.7)
Bronchoscopy unit, n (%)				
– High complexity – Offer volume reduction techniques, n (%)	39 (33.6) 11 (9.5)	0 0	6 (10.3) 0	19 (44.2) 11 (25.6)
Diagnostic tests for alpha 1 antitrypsin deficiency (AATD), n (%)				
– AATD genotyping – Sequencing	61 (52.6) 16 (13.8)	9 (60) 2 (13.3)	23 (39.7) 2 (3.4)	29 (67.4) 12 (27.9)
Consulting pulmonologist, n (%)	67 (57.8)	11 (73.3)	34 (58.6)	22 (51.2)
Therapeutic education programme, n (%)	40 (34.5)	3 (75)	14 (87.5)	23 (95.8)
Discharge follow-up and support programme, n (%)	64 (55.2)	9 (60)	28 (50)	27 (64.3)
Consulting specialist for COPD, n (%)	53 (45.6)	5 (35.7)	20 (35.1)	28 (65.1)
COPD process protocols written, n (%)	42 (36.2)	0	15 (60)	27 (77.1)
Unscheduled consultation for immediate COPD care, n (%)	51 (44)	5 (33.3)	22 (37.9)	24 (55.8)
Specialised COPD consultation, n (%)	61 (52.6)	1 (6.7)	25 (43.1)	35 (81.4)
– Accredited	18 (30)		4 (16)	14 (40)
Time allocated to consultation (minutes), median (IQR)				
– First consultation – Review consultation	30 (20–30) 15 (15–20)	30 (30–30) 20 (20–20)	20 (20–30) 15 (15–17)	30 (20–30) 15 (15–20)
Nurse consultation for COPD care, n (%)	45 (38.8)	4 (26.7)	16 (27.6)	25 (58.1)
Specialised smoking consultation, n (%)	56 (48.3)	5 (33.3)	19 (32.8)	32 (74.4)

Data are represented as absolute (relative) frequencies or median (IQR: interquartile range). Abbreviations: IMCU: intermediate care unit; * number of beds assigned per nurse. Hospital complexity level I (primary hospital); hospital complexity level II (secondary hospital); hospital complexity level III (tertiary hospital); AATD: alpha 1 antitrypsin deficiency. High-complexity bronchoscopy unit: perform advanced diagnostic and therapeutic bronchoscopic procedures (endobronchial ultrasound and computed tomography navigation) with multidisciplinary operating structure; specialised COPD consultation: clinic is staffed by team of COPD experts with multidisciplinary structure that attends to patients with more complex conditions; consulting pulmonologist: consultant pulmonologists integrated in primary care work team; consulting specialist for COPD: consultant pulmonologists with COPD expertise integrated in primary care work team; discharge follow-up and support programme: multidisciplinary intervention with team of pulmonologists, physiotherapists, and nurses for COPD patient care at discharge; therapeutic education programme: structured and systematic intervention that aims to improve patients’ knowledge, skills, and motivation to improve patients’ self-management of their illness.

**Table 2:** Organisational performance of respiratory units.

	Overall	Level I	Level II	Level III
Number of discharges per pneumology unit/100,000 inhabitants, median (IQR)	266.2 (186.8–399.2)	218.3 (99.3–402.1)	250 (304.2–841.2)	294 (193.3–417.8)
Average length of stay (days), m (SD)	8.72 (1.26)	7.82 (1.17)	8.85 (1.24)	8.70 (1.26)
Number of 30-day readmissions for COPD/100 discharges *, median (IQR)	14.9 (10.1–18.7)	10.7 (8.2–15)	15.5 (12.1–19.4)	13.1 (8.6–16.7)
Number of patients on acute non-invasive ventilatory support per year/100 discharges, median (IQR)	13.8 (9.2–25)	6.2 (5.4–20)	13.8 (9.2–25)	14.4 (10.5–25.4)
In-hospital mortality #, m (SD)	4.10 (1.18)	2.6 (1.23)	4.09 (1.14)	4.27 (1.17)
Number of spirometries performed per month, median (IQR)	303 (160–450)	160 (100–280)	222.5 (135–400)	580 (350–845)
Number of spirometries per month/100,000 inhabitants, median (IQR)	121.4 (70–225.7)	170.5 (95.8–320)	104.2 (60.8–159)	141.7 (77.7–230.7)
Number of diffusion tests performed per month, median (IQR)	40 (100–175)	10 (0–40)	78 (40–135)	200 (110–300)
Number of lung volume measurements performed per month, median (IQR)	20 (45–115)	1 (0–25)	40 (20–90)	100 (35–150)
Number of 6 min walk tests per month, median (IQR)	27 (13–51)	15 (10–25)	22 (12–45)	50 (30–100)
Number of patients in a home ventilation programme, median (IQR)	150 (48.7–282)	70 (25–150)	145.2 (29–205)	260 (92–385)
Number of patients on home ventilation/100,000 inhabitants, median (IQR)	57.8 (20.3–100.5)	51.8 (15.1–115.4)	49.3 (14.5–95.2)	74.3 (25.8–99.6)
Number of bronchoscopic procedures per year, median (IQR)	468 (210–750)	86 (50–200)	321 (200–500)	763 (550–1177)
Number of bronchoscopic procedures per year/100,000 inhabitants, median (IQR)	164 (114.8–244.8)	94.9 (67.9–156.8)	156.2 (112.5–233.3)	210.8 (153.5–274)
Number of patients undergoing endoscopic volume reduction per year, median (IQR)	5 (4–17)			5 (4–18)
Number of patients diagnosed with AATD per year, median (IQR)	5 (2–20)	4 (2.7–10)	5 (2–13.5)	14.5 (3.25–50)
Number of patients diagnosed with severe AATD per year, median (IQR)	2 (1–5)	1 (0–2)	2 (0–3)	4 (1–10)
Number of patients on augmentation therapy for AATD per year, median (IQR)	2 (0–4)	1 (0–1)	2 (0–3)	3 (1–7)

Data are represented as median (IQR: interquartile range); hospital complexity level I (primary hospital); hospital complexity level II (secondary hospital); hospital complexity level III (tertiary hospital); * number of 30-day COPD readmissions/100 discharges at respiratory units; # mortality: number of deaths/100 admissions to pneumology ward. Abbreviations: AATD: alpha 1 antitrypsin deficiency.

**Table 3:** Determinants of 30-day readmission rates in patients with chronic obstructive pulmonary disease (COPD).

	30-Day Readmissions for COPD ‡	*p*-Value #	β Coefficient (CI95%)	*p*-Value #	Regression Coefficients ^α^
Hospital complexity, m (IQR)		0.041 *			
– Level I, or primary hospital – Level II, or secondary hospita – Level III, or tertiary hospital	10.7 (8.2–15.0) 15.5 (12.1–19.4) 13.1 (8.6–16.7)		2.036 (−2.524–6.596) −0.441 (−5.059–4.177)	0.378 0.850	
Availability of specialised COPD consultation, m (SD)		0.006 *			
No Yes	16.2 (5.3) 13.2 (5.2)		−2.932 (−4.984–0.881)	0.006 *	
Availability of immediate attention, m (SD)		<0.001 *			
No Yes	16.3 (5.1) 12.5 (5.1)		−3.805 (−5.792–1.818)	<0.001 *	
Availability of post-discharge follow-up programme, m (SD)		<0.001 *			
No Yes	17.7 (5.1) 12.1 (4.3)		−5.624 (−7.492–3.756)	<0.001 *	
Specialist COPD consultant available, m (SD)		0.007 *			
No Yes	15.9 (5.3) 13 (5.2)		−2.865 (−4.927–0.802)	0.007 *	
COPD nurse’s office available, m (SD)		<0.001 *			
No Yes	16.2 (5.3) 12.0 (4.5)		−4.260 (−6.266–2.253)	<0.001 *	
In-hospital stay			1.789 (1.023–2.556)	<0.001 *	0.418
Number of acutely ventilated patients per 100 discharges			0.131 (0.063–0.199)	<0.001 *	0.348
Availability of IMCU, m (SD)		0.054			
No Yes	15.5 (5.5) 13.3 (5)				
Have written COPD protocols, m (SD)		0.693			
No Yes	13.6 (5.8) 13 (5)				
Have a respiratory rehabilitation programme, m (SD)		0.146			
No Yes	15.5 (5.7) 13.9 (5.2)				
Have 24 h emergency care provided by pulmonologists, m (SD)		0.381			
No Yes	14.9 (5.7) 13.9 (4.8)				
Number of discharges per 100,000 inhabitants					−0.342
Number of pulmonologists per 100,000 inhabitants					−0.209

Data are represented as mean (standard deviation) or median (IQR: interquartile range); ‡ number of 30-day COPD readmissions/100 discharges in respiratory units; # Student’s *t* or Kruskal–Wallis; α Spearman’s rank correlation coefficient. * *p* < 0.05 (statistical significance). Abbreviations: IMCU: intermediate care unit.

**Table 4:** Multivariate analysis of 30-day readmissions in patients with COPD.

Variable	β Coefficient	Lower 95% CI	Upper 95% CI	*p*-Value
Availability of immediate attention	−1.17680	−3.2926682466	0.9390732	0.217624
Availability of therapeutic COPD education programme	−1.06925	−3.2966721762	1.1581651	0.323483
Availability of specialised COPD consultation	−0.27570	−2.4957277620	1.9443309	0.882982
Average length of stay	1.21028	0.4459950155	1.9745690	0.004149
Availability of consulting COPD specialist	−1.00004	−3.2838274923	1.2837502	0.279671
Number of patients on acute non-invasive ventilatory support per year/100 discharges	0.07243	0.0065636954	0.1383044	0.077775
Availability of post-discharge follow-up programme	−3.89691	−5.8584889147	−1.9353364	0.000231

## Data Availability

Dataset available on request from the authors.

## Supplementary Materials

The following supporting information can be downloaded at https://www.mdpi.com/article/10.3390/healthcare13030317/s1, Supplementary S1: Participants in COPD Observatory study; Supplementary S2: Hospital-related variables; Supplementary S3: Determinants of in-hospital mortality.

## Abbreviations

The following abbreviations are used in this manuscript:

IMCU	Dependent intermediate care unit
COPD	Chronic obstructive pulmonary disease
SEPAR	Spanish Society of Pneumology and Thoracic Surgery
