# Supplementary material for: Resources and Readmission for COPD Exacerbation in Pneumology Units in Spain: The COPD Observatory Project
Source: Healthcare (Basel). 2025 Feb 4;13(3):317. doi: 10.3390/healthcare13030317 (PMC11817094; doi:10.3390/healthcare13030317)
Supplement: Supplementary file 1 [file healthcare-13-00317-s001.zip › Supplementary S1.pdf]

Supplementery S1: Participants in COPD OBSERVATORY study

Andalucía: Ruth Ayerbe García, H. Universitario Virgen de Macarena. A Soto Venegas, H. San Juan De la Cruz, Ubeda. Armando Falces Sierra, H De La Línea, Cádiz. Ana Fulgencio Delgado. H. Infanta Elena, Huelva. José Calvo Bonachera, H Torrecardenas. Elisabeth Castillo Diaz, H. Poniente, Almería. Ana Fulgencio Delgado, H. Infanta Elena, Huelva. José Cebrián Gallardo, H. Costa del Sol, Málaga. Francisco Casas Maldonado, H. Universitario Clínico San Cecilio, Granada. Francisco cabello rueda, H. Serranía Ronda, Málaga. Francisco Campos Rodriguez, H. Universitario De Valme, Sevilla. Del Castillo Otero, H. Puerto Real, Cádiz. Eva Vázquez Gandullo, Hospital Universitario Puerta del Mar, Cádiz. Soto Campos, Hospital Universitario de Jerez de la Frontera, Cádiz. Linares Serrano, H Hare Guadix, Granada. Eva Vázquez Gandullo, H. Universitario Puerta del Mar, Cádiz. José Luis Velasco Garrido, Complejo hospitalario Universitario Virgen De La Victoria De Málaga. Cruz Molina, H. San Agustín, Jaén. Dorado Galindo, H. Regional Universitario de Málaga.

Aragón: M. Ángel Santolaria López, H Royo Villanova. Luís Borderías Clau, H General Universitario San Jorge. Cristina Aguilar Paesa, H. Clínico Universitario Lozano Blesa. José Arnaldo Grajeda Juárez, Hospital Ernest Lluch, Calatayud. Virginia Moya Álvarez, H. Clínico Universitario Lozano Blesa. Clara Viñado Mañes, H. Barbastro.

Asturias: Marta García Clemente, H. Universitario Central de Asturias. Roberto Fernández Mellado, H de Cabueñes. Susana Filgueira Martínez, H. de Jarrio. Ana Paramá Fontenla, Hospital Vital Álvarez-Buylla.

Baleares: Antonia Fuster Gomila, H Universitario Son Llazer.

Canarias: Carlos Cabrera, Hospital Universtiario de Gran Canaria Dr. Negrín, Las Palmas.

Cantabria: Carlos Antonio Amado Diago, H. Universitario Marqués de Valdecilla, Santander.

Castilla y la Mancha: José Luis Izquierdo Alonso, H. Universitario de Guadalajara. Encarnación López Gabaldón, Complejo Hospitalario de Toledo.

Castilla y León: Soledad Serrano Peña, H Universitario de Burgos. Andrea Crespo Serrano. Complejo Asistencial Universitario de Plasencia. Luis Carazo, H. Universitario Asistencial de León. Lorena Martínez Romero, H. Santa Barbara, Soria. Ignacio Lobato Astiárraga, Complejo Asistencial de Ávila.

Cataluña: Antonia Llunell Casanovas, Consorci Sanitari de Terrassa. Juan María López, H. de L'Esperit Sant. Ester López Cadenas, H Viladecans. Xavier Aguilar Bargalló, H. Universitario Joan XXIII Tarragona. José Sanz Santos, H, Mutua de Terrasa. Salud Santos Pérez, H. Universitario de Bellvitge. Joan Serra Batlles, H. Universitario de Vic. Jessica González Gutiérrez, H. Universitario Arnau de Vilanova de Lleida. Carles Sabadell, H. de Figueres, Girona. Silvia Molina, H. de Campdevañol, Girona. Carmen Santiveri, H. Dos de Mayotia, Barcelona. Nestor Soler, H. Clinic, Barcelona. Mario Serrano, H. Mollet. Elizabeth Arellano, Parc Sanitari Sant Joan de Déu (H. de Sant Boi). María del Pilar Ortega Castillo, H. de Mataró. Mónica Guerrero, Consorcio Sanitario de L'Anoia, Barcelona. Eduardo Valverde Forcada, Hospital Sant Jaume de Calella, Barcelona. Oscar Bernadich, H. Universitario de Manresa. Elena de Miguel Campos. Hospital Moisés Broggi Sant Joan Despí. Barcelona. Noelia Pablos Mateos. H. Sant Joan de Déu de Martorell.

Extremadura: Jaime Corral Peñafiel, Complejo Universitario de Cáceres. Antonio Manuel Pérez Fernández, Complejo Hospitalario del Área de Salud Mérida. Francisca Lourdes Márquez, H. Universitario de Badajoz. José Carlos Serrano Rebollo, H. de Llerena Zafra, Badajoz.

Galicia: Cristina Represas Represas, H. Álvaro Cunqueiro, Vigo. Adolfo Baloira Villar, Complejo Hospitalario Universitario de Pontevedra. Dolores Corbacho Abelaira, H. Rivera Povisa, Pontevedra. Rafael Golpe Gómez, Hospital Universitario Lucus Augusti.

Madrid: M. Isabel Andrade, H. Infanta Leonor. Soledad Alonso Viteri, H Universitario de Torrejón. Juan Luis Rodriguez Hermosa, H. Clínico San Carlos, Guillermo Doblaré Higuera, H Universitario Infanta Elena. Carlos Almonacid Almansa, H Universitario Puerta de Hierro, Majadahonda. José Ramon Donado Uña, H. Universitario de Fuenlabrada. David Jiménez Castro, H. Universitario Ramón y Cajal. Raquel Casitas Mateo, H. Universitario La Paz. José Miguel Rodríguez González-Moro, H. Príncipe de Asturias, Alcalá de Henares. Mª Ruth Herrero Mosquete, H. Universitario Infanta Cristina. Araceli Abad, H. Universitario Getafe. Mercedes Izquierdo Patrón, H. Universitario Fundación Alcorcón. Tamara Alonso Perez, H. Universitario La Princesa. Luis Puente Maestu, H. Universitario Gregorio Marañón.

Murcia: Elena Paya Peñalver, Hospital comarcal del Noroeste. Juan Miguel Sánchez Nieto, H Morales Meseguer. Pedro Méndez Martínez, H. General Universitario Reina Sofia, Murcia. Elena Paya Peñalver, H. Comarcal del Noroeste. José Andujar Blesa, H. Universitario Virgen de Arrixaca, Murcia. Antonio Santacruz Siminiani, H. Santa Lucia, Cartagena. Carmen Fernández Sánchez, H. Rafael Méndez.

Navarra: Pilar Cebollero Rivas, H. Universitario de Navarra. Juan Pablo De Torres, Clínica Universitaria de Navarra.

País Vasco: Itxaso Sagayo Reza, H Bidasoa. Cristobal Esteban González, H Galdakano. Milagros Iriberri Pascual, H. Universitario De Cruces. Susana Chic Palacín, H. de Mendaro, Jesús Camino Buey, H. Sant Eloy de Barakaldo.

Rioja: Carlos Ruiz Martínez, H. Universitario San Pedro de Logroño.

Valencia: Luis Miravet, H. Universitario La Plana. Juan Gil Carbonell. H. General Universitario Dr. Balmis. Adela Sanchis Martínez, H Marina Baixa. Eusebi Chiner Vives, H Universitario San Juan, Alicante. Jaime Signes-Costa Miñana, H. Clínico Universitario, Valencia. Estrella Fernández Fabrellas, H. universitario de Valencia. Beatriz Amat Humaran, H. Universitario del Vinalopó, Alicante. Marta Ballester Canelles, H. General de Requena, Valencia. Juan José Soler Cataluña, H. Arnau de Vilanova, Valencia. Mª Carmen Ferrando Siscar, H. Virgen de los Lirios, Alicante. Elsa Naval Sendra, H. Universitario de Alzira, Valencia. Ana Ferrando Cabida, H. Universitario La Fe. Eleuterio Llorca Martínez, H. General Universitario de Elda, Alicante.
