# Supplementary material for: Resources and Readmission for COPD Exacerbation in Pneumology Units in Spain: The COPD Observatory Project
Source: Healthcare (Basel). 2025 Feb 4;13(3):317. doi: 10.3390/healthcare13030317 (PMC11817094; doi:10.3390/healthcare13030317)
Supplement: Supplementary file 1 [file healthcare-13-00317-s001.zip › Supplementary S2- Hospital-related variables.pdf]

Supplementary S2: Hospital-related variables

	Definitions and Observations	Response Options	Observations / Proposals
Questionnaire	The OBSERVATORIO EPOC database is a registry by SEPAR, aimed at providing updated information on the pulmonology services and units within the National Health System. It currently does not include specific pediatric pulmonology units		
0. Hospital	General Data Related to the Hospital		
0.1. Autonomous Community	A: Andalusia; AR: Aragon; AS: Asturias; IB: Balearic Islands; CA: Canary Islands; CAN: Cantabria; CLM: Castilla-La Mancha; CyL: Castilla y León; CAT: Catalonia; EX: Extremadura; GA: Galicia; LR: La Rioja; CAM: Community of Madrid; MU: Murcia; NA: Navarra; PV: Basque Country; CV: Valencian Community	Complete. If unknown, extract from the CNH	
0.3. Hospital Name	Name as listed in the CNH		
0.4. Installed Beds	Information provided by the CNH		
0.5. Hospital Complexity Level	Level I Hospital: Primary**:** Few specialties, mainly internal medicine, obstetrics-gynecology, pediatrics, and general surgery, or just family medicine. Bed capacity: 30-200 beds		
	Level II Hospital: Secondary**:** 5 to 10 medical specialties; bed capacity of 200-800 beds; often referred to as a provincial hospital		
	Level III Hospital: Tertiary**:** Highly specialized equipment and staff; bed capacity: 300-1500 beds		
1. Unit Typology			
1.1. Institutional Name of the Pulmonology Service or Unit	Please indicate the option that best fits your situation	Institute or Clinical Management Area	
		Service	
		Section	

		No own organizational entity (e.g., one or more pulmonologists within the Internal Medicine Service or Unit)	
1.2. Population of the Health Area or Hospital's Area of Influence	Refers to the number of inhabitants assigned to the hospital's direct area of influence. The area of influence for reference units (e.g., sleep disorders) may be larger	Number of inhabitants	
1.3. Integration into an Assistive Network	**Assistive Network**: Integration of various resources (home, health center, local hospital, reference services, convalescence units, etc.), providing assistance with the most appropriate service (home support, consultation, day hospitalization, conventional hospitalization, surgery, medium or long-term stay units, home hospitalization, etc.) to ensure quality, continuity, and comprehensive care in the most efficient manner. An assistive network should:<br><br>• Have a defined geographical and population framework for each process block.<br>• Understand the relationship (referral criteria, discharge, and joint management) and characteristics of the integrated resources (care units).<br>• Provide instruments that ensure continuity of care (protocols, pathways, integrated care processes), known and used by healthcare professionals.<br>• Integrate teams and professionals into the functional (especially information systems) and clinical aspects (process management, disease management).	No / Yes	
1.3.1. If 1.3. Yes, Number of inhabitants in the network's area of influence		Number of inhabitants in the network's area of influence	

1.3.2. If 1.3. Yes, Names of the hospitals integrated into the network		Unidades (nombre de hospitales) integradas en la red (mencionar):	
1.4. Do the Primary Care Teams (PCT) within the hospital's area of influence have an assigned pulmonologist from the unit?	Answer 'Yes' only if each PCT has an assigned pulmonologist or if at least one monthly meeting session of the reference pulmonologist with the PCTs is held. PCT can have different denominations in various Autonomous Communities	No / Yes	
1.5. Total Number of Pulmonologists	All pulmonologists in the Service or Unit (the distribution among functional units is detailed later). Include any temporary and interim staff performing their activities in the service/unit. Do not include residents, voluntary assistants, fellows, etc. Use full-time equivalents (FTE) if necessary. For example, if a staff of 24 pulmonologists has had a reinforcement of 1 pulmonologist for 6 months, it will be calculated as 24 + 0.5 = 24.5 pulmonologists	Number of Pulmonologists	
1.6. Total Number of Pulmonology Residents		Number of Residents	Do not include residents from other specialties, nor fellows or volunteers
1.7. Name of the Unit Head			
2. Clinic	Refers to the activity of outpatient consultations, hospitalization (including critical care), pulmonary function laboratory, and bronchoscopy examinations		
2.1. Personnel			
2.1.1. Pulmonologists (ward, critical care, outpatient consultations, laboratory, bronchoscopy, etc....)	It does not include residents, volunteer assistants, fellows, research, etc. If there is no allocation of personnel by functional units, do not answer this question	Number of pulmonologists assigned to clinical activities	

2.2. Number of operational conventional hospitalization beds assigned to the unit	In case there are no beds specifically assigned to the unit, put 0. This does not include critical care beds.<br>Conventional Hospitalization. Admission of a patient with acute or chronically exacerbated pathology in a nursing unit organized and equipped to provide Level 0 and 1 care and assistance 24 hours a day to patients, where the patient stays for more than 24 hours.<br>Level 0: Patients whose needs can be met in a conventional acute hospital ward. Level 1: Patients at risk of deterioration or coming from a higher level of care, whose needs can be met in conventional hospitalization with advice and support from the critical care team. An example of Level 1 care is conventional hospitalization beds equipped with telemetry.	Number of conventional hospitalization beds	Divide by levels of care
2.2.1 Level 0 beds.	Level 0: Patients whose needs can be met in a conventional acute hospital ward	Number of Level 0 beds	In units that have the capability for central monitoring for all beds (Level 1), estimate the number of beds that, on an annual average, do not have monitoring
2.2.2 Level 1 beds.	Level 1: Patients at risk of deterioration or coming from a higher level of care, whose needs can be met in conventional hospitalization with advice and support from the critical care team. An example of Level 1 care is conventional hospitalization beds equipped with telemetry (intermediate care beds)	Number of Level 1 beds	In units that have the capability for central monitoring for all beds (Level 1), estimate the number of beds that, on an annual average, have monitoring
2.3. Number of discharges per year	Only for units with assigned conventional hospitalization beds. Indicate the data corresponding to the year 2022	Number of discharges in conventional hospitalization beds	
2.3.1 Number of COPD DRG discharges per year	Indicate the data corresponding to the year 2022		

2.4. Average length of stay	Only for units with assigned conventional hospitalization beds. Indicate the data corresponding to the year 2019	Expressed in days and with one decimal point (for example: 5.4 days)	
2.4.1 Average length of stay in COPD DRGs	Indicate the data corresponding to the year 2022		
2.5 Number of readmissions within 30 days for COPD	Indicate the data corresponding to the year 2022		
2.5 Mortality	Indicate the data corresponding to the year 2022		
2.6. Number of first consultations per year	Includes "high resolution" consultations. Indicate the data corresponding to the year 2022	Number of first consultations	Include, if applicable, the consultations of monographic units, whose data is collected in section 3 of the questionnaire, as well as, if applicable, those related to functional units
2.7. Number of follow-up consultations per year	Indicate the data corresponding to the year 2022	Number of follow-up consultations	Include, if applicable, the consultations of monographic units, whose data is collected in section 3 of the questionnaire, as well as, if applicable, those related to functional units
2.8. Is there an on-site duty service for the department or unit? (referring to the entire department or unit)		No / Yes	
2.9. Is there an on-call duty service for the department or unit?		No / Yes	

(referring to the entire department or unit)			
3. Bronchoscopy Unit			
3.1. Do you have a bronchoscopy unit?	**Level 1: High-Complexity Specialized Unit.** Must be equipped with sufficient material and human resources, a stable and multidisciplinary operating structure, and the capability to carry out any type of healthcare (interventional), teaching, or research activity related to the prevention, diagnosis, and treatment of COPD. Must facilitate the consultation and referral of patients from lower-level units. The requirements to access this level, distributed in evaluable and recommendable criteria, are detailed in the Self-Evaluation Scale section.<br><br>**Level 2: Specialized Unit.** Less complex than the previous level, these units must have a minimum of their own human and material resources to guarantee specialized care to patients. Must carry out healthcare (diagnostic and therapeutic), teaching, and research activities related to the prevention, diagnosis, and treatment of respiratory pathology. Must maintain a fluid relationship with higher complexity units for the consultation and referral of more complex patients. The requirements to access this level, distributed in evaluable and recommendable criteria, are detailed in the Self-Evaluation Scale section.<br><br>**Level 3: Basic Unit.** This is the basic structure led by a specialist who dedicates a large part of their work to the techniques they are accredited for, although not necessarily exclusively, standing out for that specific work at their center. Must maintain a fluid relationship with higher complexity units for the consultation and referral of more complex patients. The requirements to access this level, distributed in evaluable and recommendable criteria, are detailed in the Self-Evaluation Scale section.	0.Does not have a Bronchoscopy Unit<br>Level 1: High-Complexity Specialized Unit.<br>Level 2: Specialized Unit.<br>Level 3: Basic Unit.	According to SEPAR criteria, even if the unit is not accredited

4.1. Number of pulmonologists assigned to the unit		Number of pulmonologists	
3.2.2. Number of diagnostic bronchoscopies	Indicate the data corresponding to the year 2022	Number of diagnostic bronchoscopies	
3.2.3. Number of therapeutic bronchoscopies	Indicate the data corresponding to the year 2022	Number of therapeutic bronchoscopies	
3.2.4. Number of interventional bronchoscopies	Indicate the data corresponding to the year 2022	Number of interventional bronchoscopies	
4.Pulmonary Function Laboratory		No / Yes	
4.1. Number of pulmonologists assigned to the unit		Number of pulmonologists	Full-time equivalents.
4.2. Number of nurses assigned to the unit	Do not include nursing assistants	Number of nurses	Full-time equivalents
4.3. Number of spirometries	Indicate the data corresponding to the year 2022	Number	If the technique is not performed, leave it blank
4.4. Number of six-minute walk tests	Indicate the data corresponding to the year 2022	Number	If the technique is not performed, leave it blank
4.5. Lung volumes (Plethysmography/helium)	Indicate the data corresponding to the year 2022	Number	If the technique is not performed, leave it blank
4.6. Diffusion	Indicate the data corresponding to the year 2022	Number	If the technique is not performed, leave it blank
4.7. Exercise test with oxygen consumption	Indicate the data corresponding to the year 2022	Number	If the technique is not performed, leave it blank

5.Intermediate Respiratory Care Unit			
5.1. Is there an Intermediate Respiratory Care Unit in your center that attends to patients with acute COPD without the direct responsibility of the Pulmonology Service/Unit?		No / Yes	
5.1.1 If yes, which specialist is responsible?	Only for those Intermediate Care Units that do not depend on the Pulmonology Service/Unit	Intensive Care Medicine	According to SEPAR criteria, even if the unit is not accredited (http://www.separ.es/areas/acreditacion-de-unidades-de-ventilacion-mecanica-no-invasiva-domiciliaria/niveles-de-acreditacion)
5.2. If you have an Intermediate Respiratory Care Unit that structurally and functionally depends on the Pulmonology Service/Unit			Si no se realiza la técnica dejarlo en blanco
5.2.1. Number of beds in the Intermediate Respiratory Care Unit			
5.2.2. Number of pulmonologists		Number of pulmonologists	

5.2.3. Number of nurses assigned to the unit	Do not include nursing assistants	Number of nurses	Full-time equivalents (if, for example, a pulmonologist spends 3 days a week on Home Noninvasive Ventilation and 2 days on general outpatient consultation, they will be counted as 0.6)
5.2.4. Number of acutely ventilated patients per year	Indicate the data corresponding to the year 2022	Number	
5.2.5. Number of patients on Home Noninvasive Ventilation	Indicate the data corresponding to the year 2022	Number	
6.Specialized consultations			
6.1. If you have a specialized COPD consultation		No / Yes	
6.1.1. Time allocated for initial consultations		Time allocated for consultation (minutes)	
6.1.2. Time allocated for follow-up consultations		Time allocated for consultation (minutes)	
6.1.3. Conducts multidisciplinary sessions			
6.1.4. Has written protocols			
6.1.5. Referral from primary care			
6.1.6. Has nursing for the COPD process			

6.1.7. There is a pulmonologist consultant for COPD			
6.1.8. Accreditation by SEPAR (level)			
6.2. If you have a Smoking Cessation Clinic			
7.Have you developed a COPD unit?	There is a defined care process	No / Yes	
7.1.1. Number of pulmonologists			
7.1.2. Number of nursing staff			
7.3. Has an "open doors" consultation		No / Yes	
7.3.1. Number of patients attended			
7.4. Has a Day Area		No / Yes	
7.4.1. Number of patients attended			
7.5. Has a nursing consultation		No / Yes	
7.5.1. Has a therapeutic education program			
8.Does your center have a Pulmonary Rehabilitation Unit that attends to COPD patients?			
8.1. Does your center have a Pulmonary Rehabilitation Unit that attends to COPD			

patients without direct responsibility from the Pulmonology Service/Unit?			
8.2. If yes, what specialty is responsible?		No / Yes	
8.3. Does your center have a Pulmonary Rehabilitation Unit that attends to COPD patients with direct responsibility from the Pulmonology Service/Unit?		No / Yes	
8.3.1.1. Number of pulmonologists in the Pulmonary Rehabilitation Unit		Number of pulmonologists	
8.3.1.2. Number of respiratory physiotherapists in the Pulmonary Rehabilitation Unit			
8.3.1.3. Number of respiratory nursing staff in the Pulmonary Rehabilitation Unit			
8.3.2. Number of COPD patients attended per year in the Pulmonary Rehabilitation Unit		Number of patients	
9.Management of the COPD process		Number of members of the service with a doctoral degree	Includes all personnel

9.1. Are there multidisciplinary meetings of the care team? If so, how many per quarter?		No / Yes	
9.2. There is a Specialist Consultant for the COPD process		No / Yes	
9.3. A program of clinical/training sessions has been established in Primary Care. If so, how many per quarter?	Regular meetings of the Service/Unit team with Primary Care in their reference population	No / Yes	
9.4. Is there a follow-up and support program for discharge from the Pneumology Service/Unit?		No / Yes	
10.Care for AATD (Alpha-1 Antitrypsin Deficiency)	Alpha-1 Antitrypsin Deficiency		
10.1. What tests are available for the diagnosis of AATD?			
10.1.1 Measurement of AAT in blood		No / Yes	
10.1.3 Phenotype		No / Yes	
10.1.4 Genotyping in blood		No / Yes	
10.1.5 Progenika: dry blood spot or oral smear		No / Yes	
10.2. Number of patients with AATD		Number of patients	

attended to annually in your service			
10.2.1 Number of severe AATD cases	Severe AATD is defined by AAT levels below 57 mg/dl	Number of patients	
10.2.2 Number of cases registered in the EARCO Registry		Number of patients	
10.2.3 Number of patients with AATD receiving augmentation therapy with AAT		Number of patients	
