# Supplementary material for: Resources and Readmission for COPD Exacerbation in Pneumology Units in Spain: The COPD Observatory Project
Source: Healthcare (Basel). 2025 Feb 4;13(3):317. doi: 10.3390/healthcare13030317 (PMC11817094; doi:10.3390/healthcare13030317)
Supplement: Supplementary file 1 [file healthcare-13-00317-s001.zip › Supplementary S3.pdf]

Supplementary S3: Determinants of in-hospital mortality

	in-hospital mortality#	p-valueα	Regression Coefficients*
Hospital complexity level, m (IQR)		0.082	
- Level I or primary hospital	2.6 (2.1-4.3)		
- Level II or secondary hospital	4.1 (3.2-4.8)		
- Level III or tertiary hospital	4.4 (3.6-5)		
Number of patients on acute noninvasive ventilatory support per year/100 discharges			0.622
Availability of IMCU, m (IQR)		0.275	
Not	4.1 (3.1-4.7)		
Yes	4.5 (3.1-5.1)		
Availability of pneumology beds equipped with telemetry, m (IQR)		0.842	
Not	4.3 (3.0-5.0)		
Yes	4.3 (3.1-4.8)		
Availability of 24-hour emergency care provided by pulmonology, m (IQR)		0.180	
Not	4.1 (3.1-4.7)		
Yes	4.5 (3.1-5.1)		
Availability of COPD process protocols written, m (IQR)		0.931	
Not	4.2 (3.4-4.8)		
Yes	4.1 (3.1-4.8)		
Availability of Consulting specialist for the COPD, m (IQR)		0.834	
Not	4.2 (3.0-4.9)		
Yes	4.1 (3.1-4.8)		

Availability of discharge follow-up and support program for COPD, m (IQR)<br>Not<br>Yes	 4.5 (3.1-5.1)<br>4.1 (1.9-6.7)	0.485	
Availability of Nurse consultation for COPD care, m (IQR)<br>Not<br>Yes	 4.4 (3.2-5.0)<br>4.1 (3.1-4.7)	0.314	
Availability of consulting specialist for the COPD, m (IQR)<br>Not<br>Yes	 4.3 (3.1-4.8)<br>4.1 (3.5-5)	0.702	
Availability of pulmonary rehabilitation program for COPD, m (IQR)<br>Not<br>Yes	 4.1 (3.0- 4.9)<br>4.2 (3.1-4.8)	0.613	
Number of 30-day readmissions for COPD /100 discharges			0.267
Average length of stay (days)			0.240
Number discharges per pneumology unit/100,000 inhabitants			-0.046
Number of pulmonologists/100,000 inhabitants			0.059

Footnote of Table 5: Data are represented as median (IQR: interquartile range); α T Student o Kruskal-Wallis
* Spearman's Rho correlation coefficient; #In-hospital mortality: number of exits/100 admissions to pneumology ward.
Abbreviations: *IMCU: Intermediate Care Unit* dependent;
